# Albumin adsorption on CoCrMo alloy surfaces

**DOI:** 10.1038/srep18403

**Published:** 2015-12-17

**Authors:** Yu Yan, Hongjuan Yang, Yanjing Su, Lijie Qiao

**Affiliations:** 1 Corrosion and Protection Center, Key Laboratory for Environmental Fracture (MOE) University of Science and Technology Beijing, Beijing 100083, China

## Abstract

Proteins can adsorb on the surface of artificial joints immediately after being implanted. Although research studying protein adsorption on medical material surfaces has been carried out, the mechanism of the proteins’ adsorption which affects the corrosion behaviour of such materials still lacks *in situ* observation at the micro level. The adsorption of bovine serum albumin (BSA) on CoCrMo alloy surfaces was studied *in situ* by AFM and SKPFM as a function of pH and the charge of CoCrMo alloy surfaces. Results showed that when the specimens were uncharged, hydrophobic interaction could govern the process of the adsorption rather than electrostatic interaction, and BSA molecules tended to adsorb on the surfaces forming a monolayer in the side-on model. Results also showed that adsorbed BSA molecules could promote the corrosion process for CoCrMo alloys. When the surface was positively charged, the electrostatic interaction played a leading role in the adsorption process. The maximum adsorption occurred at the isoelectric point (pH 4.7) of BSA.

The adsorption of proteins on solid surfaces has occupied a wide range of research projects due to its important role in biosensors, drug delivery systems, artificial tissues and so on^1^,^2^,^3^,^4^. For any foreign material transplanted into the body, the initial stage is the adsorption of proteins that would determine the subsequent performance of the transplant^5^. The adsorption of proteins on a solid surface is a complex process combing hydrophobic, electrostatic and hydrogen-bonding interactions. Recent studies consider whether the hydrophobic interaction or the electrostatic interaction is more important, depending on the properties of the solid surface and the surrounding environment^3^. There are abundant reports on the adsorption of various proteins on different material surfaces^2^,^3^,^4^,^5^,^6^,^7^,^8^. Adsorption is the process whereby molecules adhere to solid surfaces. Such proteins adsorption is an instantaneous process occurring on its first contact with biological fluids and tissues. Protein adsorption studies have focused on the adsorption kinetics, dynamics and thermodynamics, the spectroscopic studies of protein structure and function in the adsorbed state^5^,^6^. The nature and amount of the protein adsorption layer depends on materials surface properties, such as wettability, polar or ionic interaction, chemical structures and topography of the surface^7^. Proteins tend to unfold or denature on the surface of some materials due to the change of energy^9^. However, the denaturation of proteins on metal surface is still under investigation.

The electrostatic interaction is related to both the charge of the solid surface and the pH value of the solution that determines the net charge of the proteins. In biological systems, the pH value is around 7.4 in normal conditions. However, some conditions such as infection and healing processes may alter the pH values around the implants^9^. Moreover, pH falls as a consequence of the hydrolysis of the metal ions released from artificial joints. Therefore, systematic studies of the influence of pH on protein adsorption are necessary.

The techniques used most often to study the behaviour of protein adsorption on a solid surface include Raman Spectroscopy^10^, quartz crystal microbalance (QMC), ellipsometry, infrared spectroscopy and various electrochemical techniques^11^,^12^,^13^. Surface kelvin potential force microscopy (SKPFM) makes it possible to obtain the morphology and the corresponding surface potential at the same time. From the morphology images, the thickness and the distribution of the proteins can be obtained. In addition, SKPFM can provide insight into the interaction between proteins and the metal substrates at the micro level. SKPFM can also be used to explain the problems related to corrosion of the tested material covered with organic species.

CoCrMo alloy is widely used as a biomaterial in dental skeletal structures and hip and knee joint replacements owing to its good biocompatibility and excellent mechanical behaviour^14^. Although there have been a few studies reporting the effect of proteins on the corrosion behaviours of biomedical materials, the mechanisms through which proteins influence the corrosion reactions are still not fully understood. The main objective of this work is to elucidate the effects of the pH and the surface charge on the adsorption of albumin on CoCrMo alloy surfaces by SKPFM. A phosphate buffered saline solution with the addition of bovine serum albumin (BSA) was employed as the simulated body fluid.

## Experiment

### Material and sample preparation

The experimental material was conventional medical cobalt-chromium-molybdenum alloy, the chemical composition of which is shown in Table 1. The specimens were machined into a cylinder with a dimension of Φ20 × 5 mm. The surface of the specimen was wet ground with SiC paper up to 2000 grit, then fine polished. All specimens have a surface roughness Ra value of about 10 nm, which is consistent with the surface finish of the commercial hip replacement components. Then the specimens were cleaned in ethanol and distilled water, followed by drying with an N_2_ gas flow.

Bovine serum albumin (BSA) was purchased from Ya Qinuo Company, Beijing. The BSA structure contains 607 amino acid residues, and the molecular weight is 66.446 kDa. The isoelectric point for BSA is 4.7–4.9^15^.

### Electrochemical tests

The simulated body fluid employed in electrochemical experiments was the phosphate buffered solution (PBS), with 0.8 g NaCl, 0.2 g KCl, 1.44 g Na_2_HPO_4_ and 0.24 g KH_2_PO_4_ per litre. The PBS solutions are adjusted with the addition of diluted HCl and diluted NaOH in order to reach a pH of 3, 4.7 and 10. Then, a certain amount of bovine serum albumin (BSA) was added to the PBS solutions, resulting in a 10 g/L solution concentration of BSA for the electrochemical experiments.

Electrochemical experiments were carried out with a three-electrode cell which consists of the specimen as the working electrode (WE) (20 mm in diameter), a platinum wire as the counter electrode (CE) and a silver/silver chloride electrode as the reference electrode (RE).

### SKPFM measurements

SKPFM measurements were carried out using a dimension Nanoscope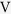, from Veeco Instruments Inc. All measurements were conducted under the tapping mode. The probes used in measurements were ANSCM-PT-50 Pt-Ir-coated silicon tips with a spring constant of 0.8–3.1 N/m and a resonant frequency of 47–76 kHz. The Pt-Ir coating is about 25 nm thick for an optimum combination of durability, conductivity and resolution. The lift mode was used to recode a second signal in addition to the surface topography. In the experiments, three different regions were scanned for every sample.

### Auger electron testing

Auger electron test was performed using a Nano scanning auger system PHI-700 (ULVAC-PHI Company, Japan), an adopted coaxial electron gun and a CMA energy analyser. The electron gun pressure was 5 kV, energy resolution was 1‰, the incident angle was 30° and the analysis room vacuum degree was better than the 3.9 × 10^−9^ Torr. The detection was based on GB/T 26533–2011: the general principles of the auger electron energy spectrum analysis method. The sputtering used a scanning type Ar gun, the prototype was the thermal oxidation SiO_2_/Si, and the sputtering rate was 6 nm/min.

## Results

### The observation of BSA and carbides on the sample

**Figure 1:**
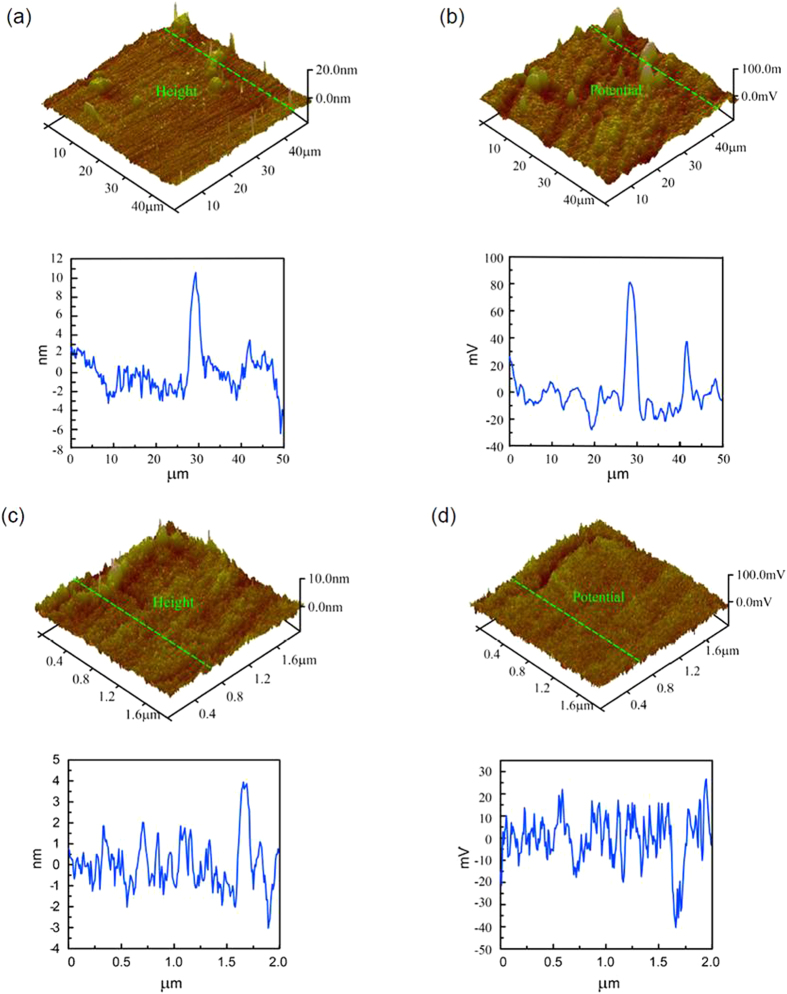
(**a**) AFM topographic and (**b**) SKPFM images of the CoCrMo alloy surface. (**c**) AFM topography and (**d**) SKPFM images of BSA on the CoCrMo alloy surface. The curves under each figure show the ups and downs of morphology and the potential of the green line on the corresponding image.

Figure 1(a) shows the topography image of the CoCrMo alloy after mechanical polishing. Some carbides (the lighter areas) are distributed randomly in the matrix, and they are about 10 nm higher than the substrate. Figure 1(b) is the SKPFM image of Fig. 1(a), which can provide an indication of the corrosion tendency. Comparing Fig. 1(a) with Fig. 1(b), the potential of carbides (the lighter areas in Fig. 1(b)) is higher than the substrate, which means that carbides are more resistant than the substrate to corrosion. Figure 1(c) is the topographical image of the BSA on the sample’s surface. The S-shaped area (the lighter area) is due to the aggregation of BSA, which is 4 nm higher than the substrate. Fig. 1(d) is the SKPFM image of Fig. 1(c). The S-shaped area in Fig. 1(d) is darker. In other words, the potential of the areas covered with BSA is lower, which indicates that adsorbed BSA can promote the electron transfer process on the sample’s surface and elevate the rate of corrosion.

### The influence of pH and voltage on protein adsorption

Because electrostatic interaction is related to both the charge of the protein molecules (which is dependent on the pH value of the electrolyte) and the charge on the metal surface, we focus on the adsorption of BSA with differently charged surfaces at pH 3.0, pH4.7 and pH10. When the pH is 3.0, 4.7 and 10, the net charges of BSA are positive, zero and negative, respectively. When −0.8 V, 0.6 V and OCP are applied, the charges of the surface are negative, positive and zero, respectively.

**Figure 2:**
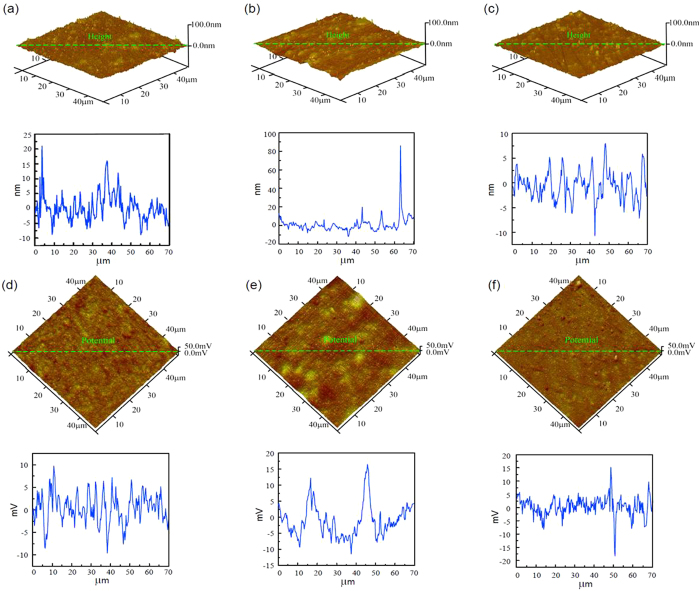
AFM topographic images of the adsorption of BSA on CoCrMo alloy surfaces at pH 3.0 (**a**), pH 4.7 (**b**) and pH 10.0 (**c**), and the corresponding SKPFM potential images at pH 3.0 (**d**), pH 4.7 (**e**) and pH 10.0 (**f**). The curves under each figure show the ups and downs of morphology and the potential of the green line on each AFM or SKPFM image.

Firstly, samples were immersed in PBS solutions with BSA for six hours, and the pH values were adjusted to 3.0, 4.7 and 10.0. Figure 2 shows the AFM topographic and SKPFM images of samples in different solutions. From the height images as shown in Fig. 2(a–c), it can be seen that there is not much difference between them (they are relatively flat, overall), except for some asperities. Similarly, the potentials of samples are uniform, as shown in Fig. 2(d–f).

**Figure 3:**
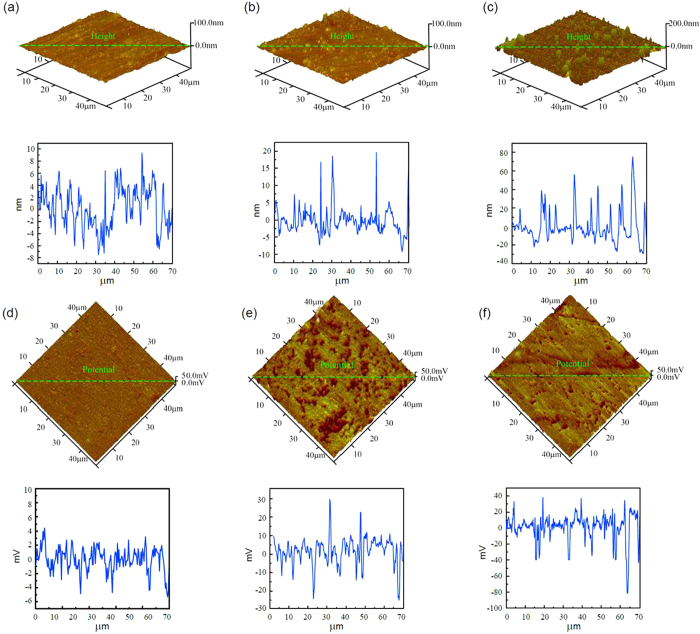
AFM topographic images of the adsorption of BSA on a CoCrMo alloy surface, with 0.6 V voltage applied at pH 3.0 (**a**), pH 4.7 (**b**) and pH 10.0 (**c**), and the corresponding SKPFM images at pH 3.0 (**d**), pH 4.7 (**e**) and pH 10.0 (**f**). The curves under each figure show the ups and downs of morphology and the potential of the green line on each image.

In order to understand the influence of the applied potential on protein adsorption, samples were used as the working electrode (WE), a platinum wire as the counter electrode (CE) and a silver/silver chloride electrode as the reference electrode (RE). A positive potential of 0.6 V (vs. Ag/AgCl) was applied to the samples for six hours. Figure 3 shows the result of AFM and SKPFM images. From the height images, it can be seen that the surface in Fig. 3(a) was the smoothest one while Fig. 3(c) was the roughest. There were many clear bumps on the sample’s surface. For the potential images, we can see that Fig. 3(e) had the highest number of low potential areas at the condition of the isoelectric point of BSA (pH = 4.7). However, compared with Fig. 3(e,f), Fig. 3(d) showed good distribution and no obviously low potential areas.

As previously mentioned, the adsorption of BSA on the surface of the sample can reduce the surface potential. The results of Fig. 3 clearly imply that the maximum adsorption of BSA occurred at pH 4.7, which was the isoelectric point of BSA. Deviating from the isoelectric point reduced the adsorption of BSA. The adsorption of BSA at pH 10 was more than at pH 3, which was due to the combined action of the charges of BSA and the sample’s surface with the competing adsorption of ions in the solution with BSA. A detailed reason for this will be given in the discussion section^1^,^4^.

**Figure 4:**
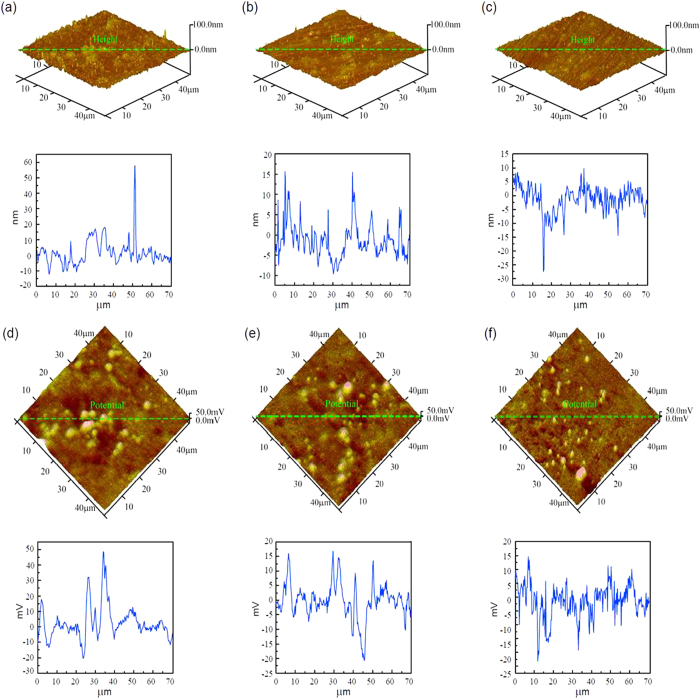
AFM topographic images of the adsorption of BSA on CoCrMo alloy surfaces at an applied −0.8 V voltage at pH 3.0 (**a**), pH 4.7 (**b**) and pH 10.0 (**c**), and the corresponding SKPFM images at pH 3.0 (**d**), pH 4.7 (**e**) and pH 10.0 (**f**). The curves under each figure show the ups and downs of morphology and the potential of the green line on each image.

AFM and SKPFM images can be seen in Fig. 4, when a potential of −0.8 V (vs. Ag/AgCl) was applied. There were some carbides on the surface at different pHs as shown in Fig. 4(a–c), whose heights were more than 20 nm. These carbides corresponded to the higher potential areas on the SKPFM pictures, which was consistent with the results in Section 3.1.

Compared with Fig. 3(e), there were fewer low potential areas at pH 4.7 (Fig. 4(e)). This was associated with the electrode reaction on the surface of the tested sample during the electrochemical test (see the discussion section). Compared with Fig. 2 and 3, the potential of the carbide was higher than that of the substrate. This was because the surface of the sample was covered with a layer of oxide in the air. However, the oxide layer dissolved when a −0.8 V potential was applied to the sample^20^,^23^, resulting in the exposure of the bare metal’s surface to the solution. The work function of the metal is lower than the oxide layer. Therefore, after peeling the oxide layer from the sample, the difference of the work function between the carbide and the substrate became greater, making the potential of the carbide higher than that of the substrate.

The surface energy of a mechanically polished CoCrMo alloy was calculated. The contact angles with three different liquids are listed in Table 2. The surface energy calculated from the contact angle of the CoCrMo alloy is about 56 Jm^−2^. From the value of the surface energy, we can see the nature of the mechanically polished CoCrMo disk is neither hydrophobic nor hydrophilic.

**Figure 5:**
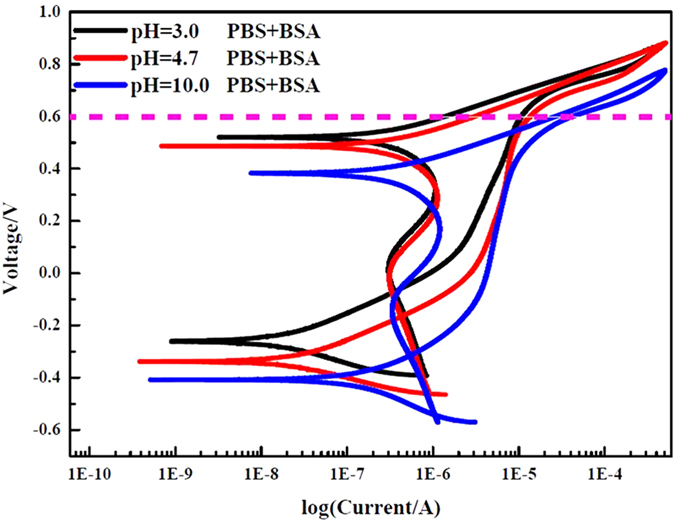
Cyclic potentiodynamic curves of CoCrMo samples in PBS solutions with BSA at pH 3.0 (black), pH 4.7 (red) and pH 10.0 (blue).

Figure 5 shows the potentiodynamic curves of CoCrMo disks in PBS solutions with BSA at different values of pH. The electrochemical parameters corresponding to Fig. 5 are shown in Table 3.

## Discussion

### The adsorption of BSA on CoCrMo alloy surfaces

The BSA molecule model is ellipsoidal with the dimensions of 4 nm × 4 nm × 14 nm^16^. According to the molecule feature, there are usually two kinds of models when the albumin monolayer is adsorbed to the material surface: side-on and end-on^17^. The thickness of the BSA-adsorbed layer showed in Fig. 1(c) is about 4 nm, which corresponds to the side-on model. Ithurbide *et al.* used XPS and a flow–cell EQCM to study the adsorption of BSA on the passivated chromium surface; they found that the layer was about 3.3 ± 0.3 nm thick, which corresponded to a monolayer of BSA^18^. Tencer *et al.* studied the BSA adlayers on Au stripes, and pointed out that the adsorbed BSA formed a monomolecular layer, likely in the side-on orientation with a thickness of about 2 nm^19^. The thickness was much lower than in our result, which was because the proteins are soft and they may be deformed by the AFM probe. However, in current AFM tests, we used the tapping mode, which can significantly reduce the effect of protein deformation. R.D.K. Misra and C. Nune^20^,^21^ studied the protein adsorption on 316 L stainless steel and reported that the self-assembly of proteins at a biointerface was governed by the grain structure because of the differences in the surface energy and the microrheological behaviour that controlled the kinetics and thermodynamics of the protein adsorption. The grain size and structure have an overriding influence on the nature of the self-assembly and the surface coverage of adsorbed proteins. The surface wettability measured as the water contact angle was about 75.14 for austenitic stainless steel^22^. However, in this study, the contact of angle of water was about 96.91 for the tested CoCrMo alloy. CoCrMo alloy had less surface energy with the same surface roughness compared with 316 L stainless steels. High surface energy provides greater driving force and preferred sites for the protein adsorption. Thus for the tested CoCrMo alloy, the AFM images suggested a more random and disordered protein arrangement because of the low fraction of the grain boundaries.

**Figure 6:**
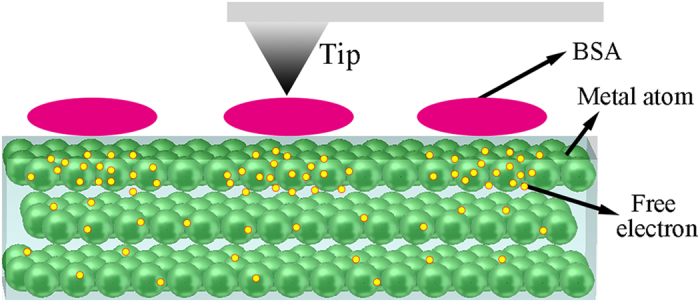
A schematic diagram of the adsorption of BSA on the CoCrMo surface which induces the gathering of free electros under SKPFM.

Surface kelvin potential force microscopy (SKPFM) is based on atomic force microscopy, which is a nulling technique. As the tip travels above the surface in the LiftMode, the tip and the cantilever experience a force wherever the potential on the surface is different from the potential of the tip. The potential is caused by the difference of the work function (the energy gap between its Fermi level and the vacuum level) between a tip and a sample. When the tip is in contact with a sample, the Fermi level of the tip will align with the Fermi of the sample, which can lead to the generation of a potential. The potential can be then monitored. The greater the work function of the sample (indicated by brighter areas on the potential images), the harder it is for electrons to be transported. Therefore, more difficult it is for materials to be corroded (corrosion reactions are the exchange process of electrons from anodic parts to cathodic parts). SKPFM can not only be used for testing conductors but can also be used for insulators. So far, there are many reports that use SKPFM to investigate proteins, DNA and organic layers^23^. For organic nanostructures adsorbing onto a conductive substrate, the measured surface potential (SP) is:


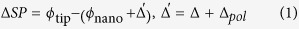


where 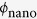 is the nanostructure work function; 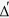 is the effective dipole barrier, which consists of two parts Δ and 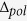; Δ is the intrinsic dipole barrier given by the interface interaction and 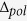 is the extrinsic dipole barrier corresponding to the polarization of the nanostructure surface resulting from the charged tip^24^. For the metal substrates covered with an organic adsorption layer, the polar parts of the molecules would attract free electrons gathering underneath the interface (as shown in Fig. 6). The potential of the CoCrMo alloy covered with BSA molecules was lower, which means that the adsorption of BSA makes it much easier for the free electrons in the metal to escape. SKPFM can be used to predict the tendency of materials to corrode. It has been found that the adsorption of BSA on a CoCrMo alloy makes the corrosion of an alloy easier, by inducing free electrons to escape. The largest amount of the adsorption (Fig. 3) and the largest I_corr_ (Table 3) – a higher I_corr_ indicates lower corrosion resistance–both occurred at pH 4.7. Many authors have performed related research before, but have reached no consistent conclusion. Yan^25^ reported that organic components could enhance the metal ion release process in the initial adsorption stage. Moulton^26^, however, pointed out the adsorbed protein can inhibit corrosion by hindering the electron transfer process. Contu^27^ concluded that serum inhibits the hydrogen evolution reaction and generates a diffusion barrier that causes anodic dissolution. Igual Muñoz^28^ suggested that albumin acted as a cathodic inhibitor that can reduce the corrosion rate. However, they all studied the influence of organics on the performance of metal corrosion at the macro level by using electrochemical methods. The process of electrochemistry is very complex. The properties of the electrolyte, the types of ions and the potential applied on the sample can all affect the results. SKPFM provides a new method to study the influence of organics on the performance of metals with reference to their corrosion behaviours at the micro level: a more accurate and intuitive method.

### The influence of pH and voltage on protein adsorption

The interaction between the surface and the protein includes: (1) the hydrophobic interaction, (2) the electrostatic interaction and (3) the hydrogen bond. It is generally believed that the essences of the three interactions are all related to the electrostatic interaction. Hydrophobic interaction occurs between nonpolar groups. Protein molecules contain polar groups and nonpolar groups simultaneously when proteins are in wet conditions. They tend to experience electrostatic attraction in polar groups, and between polar groups and water molecules, thus shoving nonpolar groups away. However, there are two opposing viewpoints: (1) the hydrophobic interaction plays the leading role in the adsorption process^29^ and (2) the electrostatic interaction is more important than the hydrophobic interaction^30^. Proteins have different charges at various pHs. The isoelectric point of BSA (pI) is about 4.7–4.9.When pH > pI, BSA is negatively charged. When pH < pI, the net charge of the BSA molecule is positive. So at pH 3.0, BSA is positively charged. At pH 10, BSA is negatively charged and at pH 4.7, the net charge of BSA is zero. The height and potential images at different pH values show no obvious differences in Fig. 2, which means the electrostatic interaction has little influence on the adsorption of BSA. It is commonly accepted that a hydrophobic surface adsorbs proteins more easily, so it is assumed that the hydrophobic interaction controls the process of the adsorption of BSA on the CoCrMo alloy surface.

Changing the pH values of solutions had little influence on the adsorption of BSA on the surface and the hydrophobic interaction played the leading role in the adsorption of BSA. However, when the surface was positively charged, the electrostatic interaction was enhanced and it became the major driving force of the adsorption. Figure 3 shows that the maximum adsorption of BSA was observed at pH 4.7 (pI). Deviating from the isoelectric point, the adsorption of BSA was reduced, and many authors have studied the effect of pH on protein adsorption with experimental results consistent with our own. BSA molecules have different structures in media with different pH values. At pH 3.0, BSA molecules show the maximum structural adaptability and can lead to the unfolding of the protein molecules^31^, which may expose the hydrophobic or hydrophilic groups according to the condition of the surface. This hydrophobic-hydrophilic interaction will lead to a decrease of adsorption. Besides, at pH 3.0, BSA molecules and the sample’s surface were both positively charged. Electrostatic repulsion between BSA and the sample’s surface would also reduce the adsorption of BSA. Finally, at pH 3.0, the concentration of H_2_PO_4_^−^ was the highest that would compete with protein molecules to adsorb on the surface and form a layer of metallic phosphates^32^. At the isoelectric point, the adsorption of BSA reached the maximum value shown in Fig. 3(b). Because the net charge of BSA molecules was zero, the lateral electrostatic repulsions between adjacent molecules can be neglected. Moreover, at the isoelectric point, BSA molecules were most compacted and could undergo the minimum conformational change^33^,^34^. At pH 4.7 there was also a layer of metallic phosphates produced on the surface due to the high concentration of H_2_PO_4_^−^ (Table 4). When the pH value increased to 10.0, BSA was negatively charged and the surface was positively charged. The electrostatic attraction between the surface and BSA molecules would enhance the adsorption. However, the repulsions between BSA molecules and the conformational change of BSA molecules decreased simultaneously. Furthermore, there were more bumps on the surface at pH 10.0 (c1) than at pH 3 (a1) and 4.7 (b1).

It can be seen from Fig. 5 that when a 0.6 V voltage was applied to the samples, the surface was still inactivate at pH 3.0 and 4.7 due to the metallic phosphates produced on the surface. However, the surface began to experience dissolution and the current began to increase at pH 10.0. According to Fig. 1, the potentials of the carbides were higher than those of the substrates, so the substrates were easier to dissolve, exposing the carbides and forming the bumps shown in Fig. 3. The largest I_corr_ occurred at pH 4.7, as shown in Table 3, owing to the maximum adsorption of BSA. According to the discussion at the beginning of the paper, BSA molecules can make it easier for the free electrons in the metal to escape to the surface, thus increasing the I_corr_.

When −0.8 V (vs. Ag/AgCl) was applied to the sample, the height and potential of the surface (Fig. 4) were different from those shown in Fig. 3. Initially, it did not show the behaviour which appeared at the isoelectric point of BSA in Fig. 3. The potential of the carbides was much higher than the substrate (Figs 2 and 3). The sample acted as the cathode when −0.8 V was applied and as the anode when 0.6 V potential was applied, so the reactions on the surface were completely different. Different reactions would make the sample’s properties different.

At any given pH, proteins have charged groups that may participate in binding them to one another or to other types of molecules. As pH drops, H^+^ binds to the carboxyl groups (COO^−^), neutralizing their negative charge, and H^+^ binds to the unoccupied pair of electrons on the N atom of the amino groups (−NH_2_) giving them a positive charge. It would not only change the net charge of the molecule (it becomes more positive) but would also result in -R groups showing ionic (electrostatic) interactions with other molecules. As pH rises, H^+^ are removed from the COOH groups, giving them a negative charge (COO^−^), and H^+^ are removed from the NH^3+^ groups, removing their positive charge. It can alter the net charge on the molecule changes (it becomes more negative).


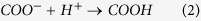



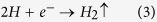



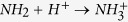


When −0.8 V was applied to the sample, the surface may undergo the following chemical reactions (Eq. (3). Owing to the reactions, the concentration of H^+^ at the surface decreased, resulting in the reaction of Eq. (2) moving to the opposite direction. In this condition, BSA was negatively charged, and the net charge of BSA was more negative than in the open circuit potential at pH 3.0 and 10.0. In this case, BSA close to the sample’s surface was negatively charged at pH 4.7. The electrostatic repulsion between the BSA molecules and between the sample’s surface and molecules would reduce the adsorption. The conformational change would also reduce the adsorption. At pH 10.0, BSA molecules were also negatively charged so the amount of the adsorption had only a minor link to the electrostatic repulsion and the conformational change. At pH 3.0, BSA molecules were positively charged, so there was an electrostatic attraction between the sample’s surface and BSA molecules that would increase the adsorption. However owing to the repulsion between BSA molecules and the conformational change, the adsorption was still not enhanced in our observation.

In the air, the surface of the CoCrMo alloy was covered with a layer of oxides. In the electrochemical tests when −0.8 V was applied to the sample, the oxides layer can dissolve slowly, exposing the bare alloy surface. The corrosion resistance of the oxides layer is excellent. The potentials of the carbides are much higher than those of the substrate, as shown by the comparison of Figs 3 and 4.

## Conclusions

AFM and SKPFM techniques were employed to characterize the adsorption and the corrosion behaviours of biomedical CoCrMo alloy in PBS solutions at different solution pHs with BSA. When the substrate was not charged, the hydrophobic effect played a leading role in the adsorption of BSA on the CoCrMo alloy surface and BSA absorbed on the surface, forming a monolayer with the side-on model. When the substrate was negatively charged, the solution pH could affect the net charge of BSA molecules resulting in a reduction of the BSA adsorption. When the substrate was positively charged, the maximum adsorption of BSA was observed at pH 4.7 (pI). Deviating from the isoelectric point, the adsorption of BSA reduced. The reasons for that are: (a) the net charge of BSA molecules was zero at the isoelectric point, so the lateral electrostatic repulsions between adjacent molecules can be neglected, and (b) BSA molecules were at the most compact form and would undergo the minimum conformational change at the isoelectric point. The adsorption of BSA can increase the corrosion rate of the tested CoCrMo alloy by inducing free electrons to escape to the surface. SKPFM also provided a new method to study the influence of organics on the performance of metal corrosion at the micro level.

## Additional Information

**How to cite this article**: Yan, Y. *et al.* Albumin adsorption on CoCrMo alloy surfaces. *Sci. Rep.* **5**, 18403; doi: 10.1038/srep18403 (2015).

## Figures and Tables

**Table 1:** The composition of the CoCrMo alloy (Wt.%).

Elements	Co	Mo	Cr	C	Ni	Si	Mn
Content	62.45	5.43	28.32	0.20	<1.0	<1.0	<1.0

**Table 2:** Contact angles of a mechanically polished CoCrMo alloy surface with different liquids, and the surface energy calculated from the contact angles.

Liquids	Contact angles (^o^)	Surface energy (Jm^−2^)
water	96.91	56.06
glycol	42.17	
formamide	61.63	

**Table 3:** Electrochemical parameters of the CoCrMo alloy in a PBS + BSA solution at different pHs.

Solutions	pH	I_corr_(A)	Ecorr(mV)
PBS + BSA	3	2.04E-8	−260
PBS + BSA	4.7	2.94E-8	−338
PBS + BSA	10	2.48E-8	−407

**Table 4:** Molar concentration of the species in the PBS solutions at different pHs.

Species	Concentration (M)		
	pH 3	pH 4.7	pH 10
H^+^	1 × 10^−3^	2.0 × 10^−5^	1 × 10^−10^
OH^−^	1 × 10^−11^	5.0 × 10^−10^	1 × 10^−4^
PO_4_^3−^	3 × 10^−16^	9.4 × 10^−13^	5 × 10^−5^
HPO_4_^2−^	7 × 10^−7^	3.7 × 10^−5^	1 × 10^−2^
H_2_PO4^−^	1 × 10^−2^	1.2 × 10^−2^	2 × 10^−5^
H_3_PO^4^	1.5 × 10^−3^	3.0 × 10^−5^	2.6 × 10^−13^
